# Hyb: A bioinformatics pipeline for the analysis of CLASH (crosslinking, ligation and sequencing of hybrids) data^☆^

**DOI:** 10.1016/j.ymeth.2013.10.015

**Published:** 2014-02

**Authors:** Anthony J. Travis, Jonathan Moody, Aleksandra Helwak, David Tollervey, Grzegorz Kudla

**Affiliations:** a Wellcome Trust Centre for Cell Biology, University of Edinburgh, Edinburgh, Scotland, United Kingdom; b Institute of Biological and Environmental Sciences, University of Aberdeen, Aberdeen, Scotland, United Kingdom; c MRC Human Genetics Unit, Institute of Genetics and Molecular Medicine, University of Edinburgh, Edinburgh, Scotland, United Kingdom

**Keywords:** CLASH, RNA–RNA interactions, Bioinformatics, High-throughput sequencing

## Abstract

Associations between proteins and RNA–RNA duplexes are important in post-transcriptional regulation of gene expression. The CLASH (Cross-linking, Ligation and Sequencing of Hybrids) technique captures RNA–RNA interactions by physically joining two RNA molecules associated with a protein complex into a single chimeric RNA molecule. These events are relatively rare and considerable effort is needed to detect a small number of chimeric sequences amongst millions of non-chimeric cDNA reads resulting from a CLASH experiment. We present the “hyb” bioinformatics pipeline, which we developed to analyse high-throughput cDNA sequencing data from CLASH experiments. Although primarily designed for use with AGO CLASH data, hyb can also be used for the detection and annotation of chimeric reads in other high-throughput sequencing datasets. We examined the sensitivity and specificity of chimera detection in a test dataset using the BLAST, BLAST+, BLAT, pBLAT and Bowtie2 read alignment programs. We obtained the most reliable results in the shortest time using a combination of preprocessing with Flexbar and subsequent read-mapping using Bowtie2. The “hyb” software is distributed under the GNU GPL (General Public License) and can be downloaded from https://github.com/gkudla/hyb.

## 1 Introduction

RNA molecules are abundant in all living cells, but, like football fans around the world, they never walk alone. Protein–RNA binding is essential for the biological function of most RNAs, and proteins accompany RNAs throughout their life cycle, from synthesis and processing to transport and degradation. Likewise, inter- or intra-molecular RNA–RNA interactions are fundamental to many processes including splicing, translation, and gene regulation. In particular, post-transcriptional regulation of gene expression mediated by miRNA molecules that base-pair with their RNA targets has been the focus of intense research efforts in recent years [1]. Other regulatory mechanisms that depend on RNA–RNA interactions include silencing of transposons by piRNAs [2], Staufen-mediated decay of mRNAs [3], and gene regulation mediated by sRNAs in bacteria [4].

The development of CLIP (Crosslinking and Immunoprecipitation) [5,6] has allowed mapping of the RNA interactomes for a variety of proteins. Several of these proteins form tripartite protein–RNA–RNA complexes, and the profiling of protein–RNA interactions can provide useful information about the corresponding RNA–RNA pairing. For example, transcriptome-wide analysis of AGO–RNA interactions in human and mouse cells has led to the discovery of many putative miRNA binding sites, a number of which could be confirmed experimentally [7–9]. Furthermore, recent CLIP studies identified various noncanonical types of interaction, such as the G-bulge sites that accounted for more than 15% of all AGO–miRNA interactions in the mouse brain [10]. However, the discovery of non-canonical interactions by CLIP is complicated by the fact that the identity of interacting partners is not observed directly and needs to be inferred bioinformatically.

We have recently described CLASH (crosslinking, ligation and sequencing of hybrids), a method for transcriptome-wide analysis of RNA–RNA interactions [11,12]. CLASH relies on purification of RNA–RNA duplexes bound to a protein of choice, ligation between the two strands of RNA duplexes to form chimeric RNAs, reverse transcription of these chimeric RNAs, high-throughput sequencing of the resulting cDNAs, and bioinformatic analysis of the sequence data to call and annotate chimeric reads. CLASH has many experimental steps in common with the CLIP and CRAC methods, but it is optimized for recovery of RNA–RNA duplexes. Here we describe the bioinformatic methods we have developed to carry out the analysis of sequencing data from CLASH experiments. We also present “hyb”, a set of command line tools that implements our approach for Unix/Linux-based computer systems. A detailed experimental protocol of CLASH will be published elsewhere (Helwak and Tollervey, in preparation).

Whilst primarily designed for the analysis of AGO CLASH data, our programs can also be used to identify chimeric reads in other applications of CLASH, as well as for the analysis of CRAC, CLIP, and RNA-Seq data. The precise interpretation of the chimeras will depend on the dataset under consideration. For example, analysis of chimeric reads in RNA-Seq experiments has recently been used to identify a novel class of RNAs with regulatory potential, the circRNAs [13,14], and most of these circRNAs can be recovered by hyb.

## 2 Overview of the method

**Fig. 1:**
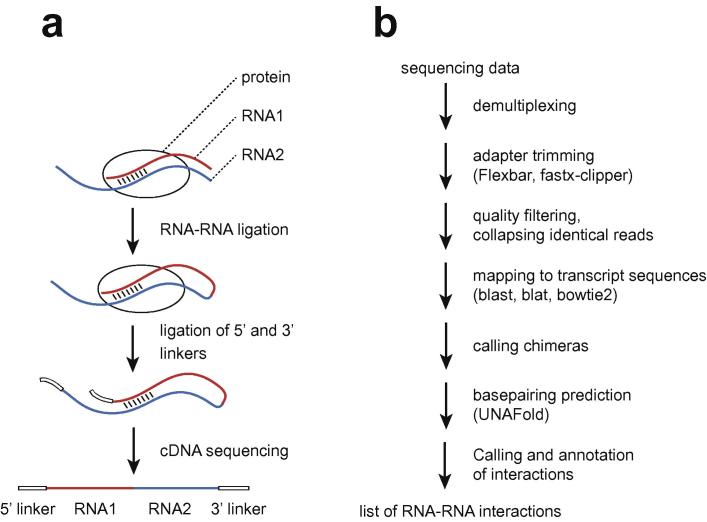
Schematic of CLASH experiment and hyb analysis pipeline.

In a CLASH experiment, interacting RNA molecules are partially digested, ligated to each other at one end, ligated to 5′ and 3′ linkers, respectively at their other end, and then subjected to single-end high-throughput sequencing. The resulting reads contain, in the following order: part of the 5′ linker, the cDNA insert, and part or all of the 3′ linker (Fig. 1a). Our bioinformatics pipeline begins by preprocessing the reads: removing linker sequences, quality filtering the data, and collapsing identical reads to speed up the computationally intensive downstream analyses (Fig. 1b).

After preprocessing, we use local alignment tools to map the reads to a custom database of transcript sequences or to the genomic sequence of the organism concerned. Reads with two non-contiguous matches to the database are identified as chimeras, whereas reads mapped contiguously are discarded. Mapping reads to transcript sequences, rather than to the genome, makes it easier to reject chimeric cDNAs generated by natural splicing events, because spliced cDNAs from mature mRNAs will generally be mapped contiguously to known mRNA transcripts.

After the chimeras are called, we fold them computationally to predict the exact pattern of basepairing between the interacting RNA molecules. Although folding algorithms cannot accurately predict the structures of long RNA molecules, they are nevertheless reliable in predicting interactions between short fragments of RNA, such as those identified by CLASH. We then use the folding predictions to annotate and classify the RNA–RNA interactions recovered.

## 3 CLASH data analysis

### 3.1 Prerequisites

**Table:** 

(a)	cDNA sequencing data in FASTQ or FASTA format.
(b)	DNA sequence of the 3′ linker used.
(c)	DNA sequences of 5′ barcodes, if present.
(d)	Database of relevant transcript sequences, or genomic sequence. A human transcript sequence database is included in our distribution. Transcript sequences can be conveniently downloaded from the Ensembl Biomart or from other specialist repositories such as mirBase or genomic tRNA database.
(e)	Computer with a Unix or Linux-based operating system conforming to POSIX (Portable Operating System Interface). The “hyb” pipeline was implemented under Bio-Linux 7 (based on 64-Bit Ubuntu 12.04 LTS) [15].
(f)	External dependencies. In addition to the internal programs developed as part of hyb, external programs are required to run the complete hyb pipeline. The complete list of dependencies is shown in Table 1, and the minimum requirements are: an adapter trimming program (FASTX toolkit or flexbar) [16,17], a read aligner (for example, bowtie2 or blast) [18,19], and an RNA folding program (UNAFold hybrid-min, or vienna RNAup) [20,21]. Many of these programs are pre-installed and supported by Bio-Linux.

### 3.2 Preprocessing reads

#### 3.2.1 Trimming of 5′ adapter sequences

**Fig. 2:**
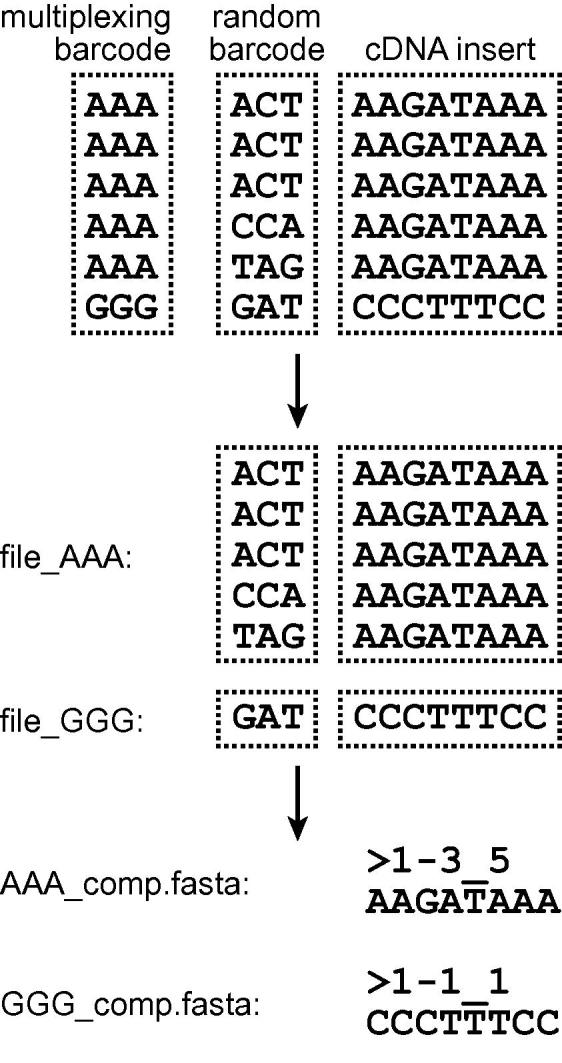
Processing of 5′ barcodes. The multiplexing parts of barcodes are used to split input data into appropriate files, whereas the random parts of barcodes are used to monitor PCR amplification artefacts. The sequence identifiers (FASTA headers) of collapsed reads are in the format “*K*-*L*_*M*”, where *K* is the frequency rank of the sequence in the input file, *L* is the number of unique random barcodes associated with the sequence, and *M* is the number of times the sequence has been found in the input file. When 5′ barcodes are absent, the identifiers of collapsed reads are in the format “*K*_*M*”.

Depending on the experimental design, 5′ linkers may contain a variable-length barcode for sample multiplexing and a random nucleotide prefix for monitoring of PCR amplification artefacts (Fig. 2). We trim the 5′ barcode allowing for 0 (default) or 1 nucleotide substitutions and no indels. Our program builds a lookup table of all the 5′ ends of reads compatible with the presence of a barcode, and then it performs one sub-string extraction and no string comparisons for each read. This allows us to process as many as 13 million reads per minute on a single CPU core. The multiplexing part of the 5′ barcode is used to split the input FASTQ format file into separate files for each sample, but is then discarded. The random part of the 5′ barcode is appended to the sequence identifiers in the FASTQ header of each read for future reference. This part of analysis can be skipped if no 5′ barcodes have been used. Typical usage for demultiplexing as a separate step is:

hyb demultiplex in=data.fastq code=barcodes.txt

where:

in=file (or in=file.gz) contains FASTQ format reads; code=file contains barcodes used to multiplex samples. "hyb" commands are normally entered on one line, but long commands can be continued onto the next line using a '\' escape character at the end of a line. The escape characters are omitted from the printed examples.

#### 3.2.2 Trimming of 3′ adapter sequences and quality filtering of reads

In a typical CLASH library, many of the recovered RNA fragments are shorter than the read length, resulting in part or all of the 3′ linker sequence to be included in the reads. Accurate 3′ linker removal is important to prevent mapping errors, but overly permissive criteria for linker recognition may result in the loss of *bona fide* genomic sequences. We have tested two linker trimming programs, flexbar and fastx-clipper, and we adopted flexbar as default. Following 3′ linker trimming, we filter the reads by insert length and by nucleotide qualities. The “preprocess” task runs this part of the analysis:

hyb preprocess qc=flexbar trim=30 len=17

in=data.fastq

where:

qc=flexbar selects Flexbar preprocessing; trim=30 trims reads from 3’ end to Phred Qual=30; len=17 specifies minimum read-length 17.

#### 3.2.3 Removal of PCR duplicates

The complexity of CLASH libraries typically ranges from a few hundred thousand to a few million cDNA molecules [11]. Following cDNA synthesis, the libraries are amplified by PCR and then sequenced, often yielding tens or hundreds of millions of reads. If the number of reads is larger than library complexity, many cDNA molecules will be sequenced multiple times. To remove potential PCR duplicates and speed up downstream analysis, we collapse identical reads while retaining information about the numbers of reads collapsed. Numbers of collapsed reads are appended to the sequence identifiers (Fig. 2). The random barcode prefixes are used to distinguish artificial PCR duplicates from mRNA fragments naturally present in the library in multiple copies. Counts of these random barcodes, if present, are also encoded in sequence identifiers of collapsed reads (Fig. 2). The reads are collapsed as part of the “preprocess” task described above. The “check” task uses the preprocessing results to analyse length distributions of uncollapsed and collapsed reads:

hyb check in=data.fastq

### 3.3 Mapping reads and calling chimeras

#### 3.3.1 Mapping of reads

In the previously published analyses, we used the blastall program to find high-scoring local matches between the reads and a database of transcript sequences [11,12]. In hyb, we implemented additional options for mapping reads using blastn (the C++ based distribution of blast), blat, pblat (parallel blat), and bowtie2. By default, hyb uses bowtie2 with the --local option to map reads against a precompiled database of human transcript sequences, included in the distribution. To create mapping databases based on a custom FASTA file, the following command may be used:

make_hyb_db genome.fasta

The default location of the reference database is specified by the HYB_DB environment variable, as described in Section 3.7. Reads can be mapped to transcriptome or genomic databases, but mapping to the genome yields a large background of chimeric reads formed by splicing, which need to be distinguished from chimeras formed by RNA–RNA ligations during the CLASH procedure. By default, hyb assumes that reads are mapped against a transcript database, and it rejects reads mapped in the antisense orientation. To accept antisense reads when mapping to a genomic sequence database, the “anti=1” option has to be set on the command line. The default sets of mapping parameters are listed in Table 2. The “detect” task performs the mapping and the chimera calling described below:

hyb detect align=bowtie2 in=data.fastq db=hOH7

where:

align=bowtie2 sets the read aligner to bowtie2; db=hOH7 selects the hOH7 Bowtie2 database.

#### 3.3.2 Identifying candidate chimeric reads

To call chimeras, we examine local matches between each read and the reference database, starting with the highest-scoring hits. We record the top-scoring match, all matches with the same mapping score as the top match, and all matches for which the gap or overlap with the previously matched area of the read is at most 4 nucleotides (default). We then identify as candidate chimeras all pairs of recorded matches with at most 4 nucleotides gap or overlap. This procedure rejects any reads with a contiguous full-length match to the database, since the overlap criterion would not be met for such reads. Short gaps or overlaps are allowed: gaps can represent post-transcriptional modification of one of the interaction partners (e.g., oligoadenylation), whereas overlaps frequently occur if one or more nucleotides in the middle of the read can be mapped to either chimeric fragment. We found that the small ambiguity in ligation sites identified, which results from such overlaps, is well tolerated and does not influence downstream analysis.

#### 3.3.3 Calling chimeric reads

The procedure above yields a list of all chimera calls compatible with the read sequence. To uniquely call each chimera, we apply a series of filters and selection criteria to this list. We select the best chimera call in the list, by considering, in turn, (1) the sum of the mapping scores of both fragments of the chimera; (2) optionally, the classes of transcripts to which the read was mapped (to prioritize miRNA–mRNA calls in the case of AGO-mediated interactions), and (3) the total number of reads mapped to each of the transcripts that form the chimera. Mapping reads to a transcript database sometimes results in ambiguous assignment to several transcripts, usually originating from the same locus. To resolve the ambiguity, we rank transcripts by the total number of mapped reads, to create an alignment reference file. We then use this reference to assign ambiguous reads to the transcript with the largest number of mapped reads. To generate consistent chimera calls across a set of related experiments, we rank transcripts according to the total number of reads across all experiments, using a common alignment reference file. The following command line illustrates the available options for identifying chimera candidates and calling chimeras:

hyb detect type=all pref=none hval=0.1 hmax=10

gmax=4 in=data.fastq db=hOH7

where:

type=all sets the search for chimeras in all classes of transcripts; pref=none do not prioritize miRNA–mRNA (mim) hybrids; hval=0.1 sets threshold e-value for hybrid fragments; hmax=10 sets maximum number of mapped locations of hybrid bits; gmax=4 sets maximum length of gap/overlap between hybrid bits.

For a test input file with 31 million reads (9 million unique reads), including >400,000 chimeras, the chimera-calling part of the analysis takes approximately 12 min on a single CPU core, and it uses 45 MB of memory, when run with default parameters.

#### 3.3.4 Bioinformatic extension of chimeric reads

At the last stage of chimera calling, for miRNA-target chimeras, we optionally extend the miRNA fragment to cover the entire miRNA sequence retrieved from our custom database, and we extend target coordinates by 25 nucleotides to make sure that the entire interaction site is included within the called region. This type of adjustment improves chimera calls for sequencing reads that do not cover the entire length of the chimeric cDNA. The same adjustment was used in the original AGO1 CLASH study [11].

hyb detect type=mim pref=mim in=data.fastq db=hOH7

where: type=mim sets the search for miRNA–mRNA (mim) hybrids and bioinformatically extend miRNA and mRNA fragments; pref=mim prioritize miRNA-mRNA (mim) hybrids.

Finally, all chimeras are written to a “.hyb” file, our gff-related format that contains sequence identifiers, read sequences, 1-based mapping coordinates, and annotation information for each chimera (Table 3).

### 3.4 In silico folding and merging of chimeras

We use the mapped coordinates of chimeras to extract the corresponding transcript or genomic sequences, and we use these sequences for RNA folding analysis. We fold transcript sequences extracted from a database, rather than read sequences, because reads may contain crosslinking-induced substitutions and deletions [22–24], which are not relevant for in vivo folding of RNA.

We use UNAFold hybrid-min with default parameters to fold RNA, and we convert the hybrid-min outputs to the commonly used dot-bracket notation, as defined in the Vienna RNA package. Optionally, the RNAup program from the Vienna package can be used for chimera folding. The dot-bracket file is then annotated with RNA sequences and coordinates to facilitate subsequent analysis.

Overlapping chimeras are then merged, similar to the bedtools merge operation [25], except that an overlap between both fragments of the two chimeras is required for merging the chimeras. This results in a list of RNA–RNA interactions, each annotated with the number of supporting chimeric reads, identifiers of supporting reads, and average folding energy. The “analyse” task runs this part of the analysis:

hyb analyse fold=UNAfold in=data.fastq db=hOH7

where:

fold=UNAfold sets hybrid-min as the RNA folding program.

### 3.5 Running the entire analysis as a single command

Each of the example “hyb” commands above can be executed separately or as one command-line for a single sample. For example the following command runs the entire analysis for a non-multiplexed dataset:

hyb preprocess qc=flexbar trim=30 len=17 min=4 check

detect align=bowtie2 word=11 analyse fold=UNAfold

in=data.fastq db=hOH7 id=bow_flex_UNA

Hyb evaluates at runtime which tasks need to be performed for a given analysis, and it uses default parameter values where needed. The same analysis as above can be run using the minimal command line below:

hyb analyse in=data.fastq db=hOH7

It is possible to run the entire “hyb” pipeline on a multiplexed sample file using the barcode file to identify each sample, as follows:

hyb demultiplex code=barcodes.txt preprocess

qc=flexbar trim=30 len=17 min=4 check detect

align=bowtie2 word=11 analyse fold=UNAfold

in=data.fastq db=hOH7

It is also possible to reuse the mapping data from a previous run of hyb, and run only the chimera calling part of the pipeline:

hyb analyse in=data_comp_hOH7.blast format=blast

align=none id=new

### 3.6 Further analysis of chimeras

The main outputs of hyb are: a file with coordinates of all the chimeras (“.hyb”), a file with the folding analysis of chimeras (“.viennad”), and a number of intermediate files that can be used when re-running parts of the pipeline with varying parameters, and for troubleshooting. Output file formats are described in the hyb documentation. The output files can be used to further analyse the patterns of RNA–RNA interactions. For example, in a previous study, we have used information from the “viennad” files for clustering of miRNA–mRNA interactions, and the information from the “hyb” files to analyse the sequence motifs found in the targets of particular miRNAs [11].

To facilitate downstream analyses, we provide several standalone scripts: hyb_merge for clustering chimeras, hyb2gff for converting a “hyb” file to the commonly used “gff” format (splitting each chimera into two entries), and enst2genome for converting from transcript to genomic coordinates. We refer the reader to the hyb documentation for additional information about these scripts.

### 3.7 Configuring environment variables

The execution of hyb can be configured using ‘environment’ variables in the Unix/Linux interactive command-line ‘shell’ (e.g., “bash”) used to invoke the program. By default, hyb sets the environment variables internally, relative to the directory “HYB_HOME” where hyb has been installed. For example, the default location for hyb data files is “HYB_DATA” and the directory containing all the alignment databases is “HYB_DATA/db”. If a user prefers to use their own database “my_db” in their own folder “my_db_dir”, they can override the defaults using e.g.:

HYB_DB=~/my_db_dir hyb in=reads.txt db=my_db

id=my_analysis analyse

In this case, the “HYB_DB” variable is only set for a single hyb run as specified on the command-line. The environment variable can also be ‘exported’ to the shell environment for all hyb runs done during the same login session using e.g.:

export HYB_DB=~/my_db_dir

hyb in=reads.txt db=my_db id=my_analysis analyse

Alternatively, the hyb environment variables can be set automatically by adding the ‘export’ statements to a start-up file that is read at by the shell at login (e.g., .profile when using “bash” under Bio-Linux). A full list of the environment variables is available in the hyb documentation and can be obtained after installing hyb using the Linux “man” command:

man hyb

### 3.8 Implementation

Hyb is implemented as an executable ‘Makefile’ that orchestrates the execution of a number of internal and external programs such as the read aligners and RNA folding software. Internal scripts are written in Perl, Python and Awk. Although designed for managing the compilation of computer software from large and complicated source-code contained in many different files, GNU make is well-suited to the requirements of bioinformatics analysis, which involves the manipulation of large text files and their transformation into other formats. Many bioinformaticians are familiar with software development and routinely use Makefiles, which capture the provenance of their analysis. Makefiles consist of goals (or tasks) and rules about how to create one file from another according to its dependencies or prerequisites. The hyb pipeline was developed as a Makefile and could be invoked using the GNU “make” command:

make -f hyb

To create an executable Makefile, the following line was used as the first line in a plain-text file:

#!/usr/bin/make -rRf

This invokes make with no built-in rules (-r) or built-in variables (-R) on the current script file (-f). The rest of the script is a conventional Makefile, but it is not necessary to invoke make explicitly on the command-line to run it. Although a minor change from the way that Makefiles are conventionally used, this makes hyb considerably more flexible and easier to parameterise.

Make has been used in other bioinformatics pipelines. For example, the “PredictProtein” server [26] invokes Make programmatically by a Perl driver script to process jobs submitted via a web GUI interface. This is done primarily because make is able to utilise the available server resources efficiently by evaluating a dependency graph according to rules specified in the Make programming language and running any independent tasks it identifies concurrently. In addition, Make will minimise the amount of unnecessary work by tracking the time and date stamps of dependencies. This has been used to great effect in the parallel short-read assembler “ABySS” [27]. The ABySS paired-end assembler is, in fact, and executable Makefile.

## 4 Parameter optimization

### 4.1 General considerations

We used the published AGO1 CLASH data [11], available on GEO as GSE50452, to benchmark hyb and establish parameter sets that maximize the total number of interactions recovered, minimize recovery of false positive miRNA–mRNA interactions, and reduce the overall time of analysis. In the context of our analysis, false positives might arise both from calling of non-chimeric reads as chimeric, and from the recovery of chimeric reads that do not represent RNA–RNA interactions. The latter could result, for example, from splicing, trans-splicing, reverse transcriptase template switching, or chromosomal rearrangements. Because it was difficult to obtain a representative dataset of true positive interactions, we adopted a computational approach to distinguish true from false positives. Specifically, we assume that true positive miRNA–mRNA interactions can be distinguished from false positives by the following characteristics:

**Table:** 

(1)	Average predicted folding energy of chimeras (stronger folding expected in true positives);
(2)	Fraction of miRNA–mRNA chimeras with seed match between miRNA and mRNA (larger fraction expected in true positives).
(3)	Fraction of chimeras with no gap or overlap between the two fragments (larger fraction expected in true positives).
(4)	Upregulation of the target mRNA following experimental depletion of the corresponding miRNA.

### 4.2 Recovery of simulated fusion and nonfusion reads

We constructed an in silico microRNA–mRNA fusion dataset to evaluate the performance of hyb. 10,000 30-nucleotide segments of mature mRNAs were extracted from Refseq and concatenated with mature microRNA sequences from miRBase. 84–91% of chimeras were recovered by hyb using default parameters depending on the aligner used. Blastn recovered the fewest, and pblat the most chimeras. Most reads not recovered were seen to align to more than 10 entries so were excluded by hyb; this is due to multiple transcripts for some genes being present in the database. Additionally, several mRNA fragments had sequences flanking the breakpoint which matched the end of the microRNA they were concatenated to, giving the alignments a larger overlap than allowed by hyb with default settings.

Conversely, when 50-nucleotide segments of mature mRNAs from Refseq were analysed, no chimeras were called. This was expected, since the 50-nucleotide segments are mapped contiguously to mRNA sequences in the database.

### 4.3 Optimization of preprocessing parameters

**Fig. 3:**
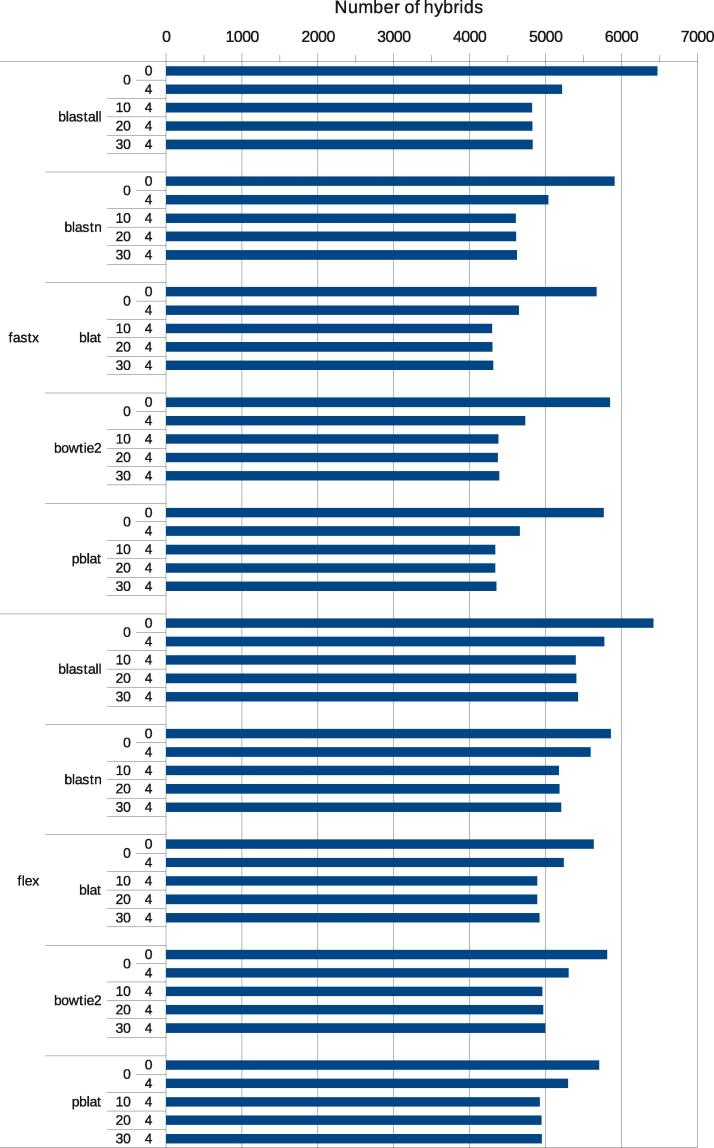
Benchmarking of preprocessing and mapping parameters. Numbers of miRNA–mRNA chimeras recovered from the E6 test dataset (from Ref. [11]) as a function of preprocessing and mapping parameters. The following parameters were explored: the choice of adapter trimming program (flexbar or fastx-clipper), the choice of mapping program (blastall, blastn, blat, pblat, bowtie2), the base quality threshold (0, 10, 20, or 30), and linker length threshold (4, or 0 which indicates no linker trimming).

We first aimed to establish optimal parameters for read preprocessing by comparing two read trimming programs (fastx-clipper and flexbar), with a range of 3′ adapter (linker) length and base quality thresholds. Flexbar with a 4-nucleotide linker length cutoff and base quality cutoffs between 0 and 30 gave reliable results. Fewer chimeras were recovered using fastx-clipper with low linker length cutoffs (Fig. 3), probably because transcript sequences were mis-identified as linkers and truncated. Conversely, up to ten percent more chimeras were found when the linker removal step was omitted. However, such excess chimeras were typically incorrect, as evidenced by their significantly weaker predicted folding (not shown), and by the fact that mapped regions of such chimeras frequently extended into the linker. Incorrect chimeras were also observed when the linker removal criteria were too stringent. Trimming reads to remove low-quality bases at the end reduced the numbers of chimeras by a few percent, but it did not increase the mean folding energy of chimeras detected.

### 4.4 Optimization of mapping parameters

**Fig. 4:**
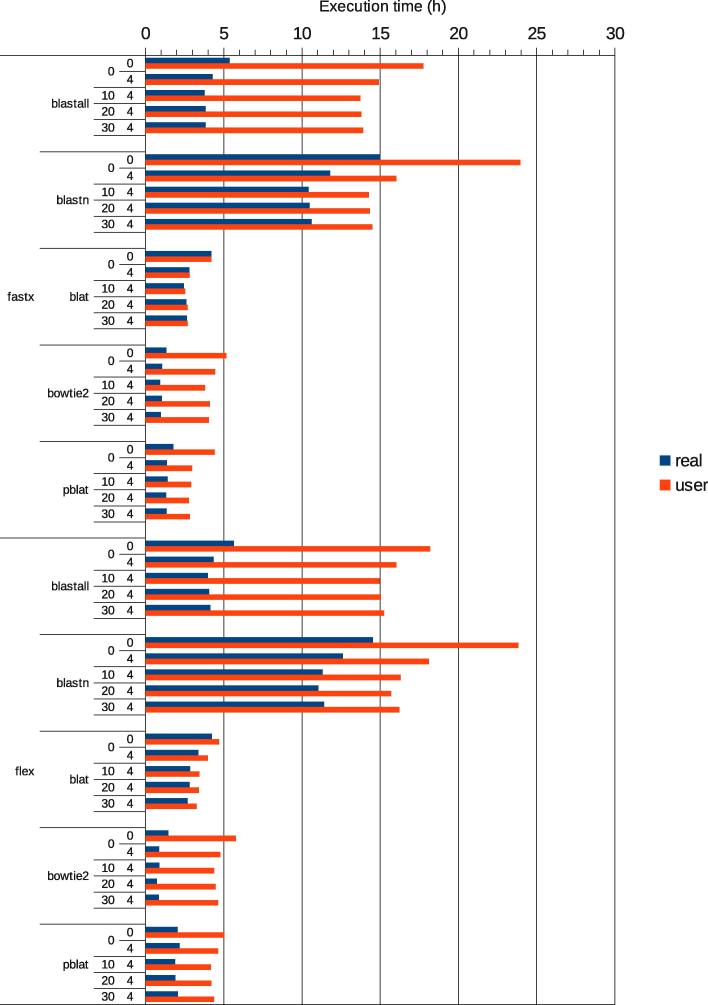
Effects of preprocessing and mapping parameters on analysis times. Data and parameter choices as in Fig. 3.

**Fig. 5:**
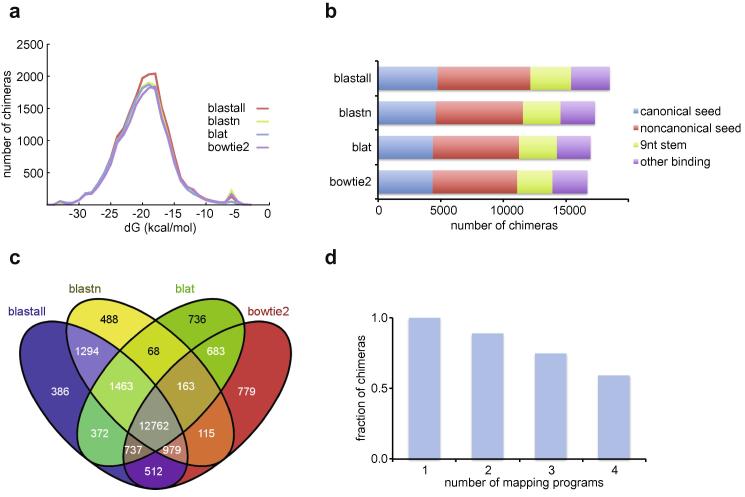
Characteristics of chimeras recovered as a function of the mapping program used. (a) Distribution of folding energies of miRNA–mRNA chimeras identified with blastall, blastn, blat, and bowtie2. (b) Types of RNA–RNA interactions recovered with each mapping program. (c) Numbers of chimeras recovered with different combinations of mapping programs, analysed with VENNY [29]. A total of 12762 interactions are found with all four mapping programs, whereas 21537 interactions are found with at least one of the programs. (d) Fractions of chimeras recovered with one or more, two or more, three or more, and four mapping programs, respectively. Analyses were performed on dataset E4 (Ref. [11]), with the following parameters: trim = 0 filt = 0 min = 4 len = 17.

The choice of the mapping program had a large effect on the time of analysis (Fig. 4). For a test dataset of ∼34 million reads, including ∼100 thousand chimeras, total hyb analysis times ranged from less than one hour to 15 h in wall-clock time. Bowtie2 was the fastest mapping program, followed by pblat, whereas blastn and blastall were slowest. However, blastn and blastall each recovered 5–10% more chimeras than bowtie2. To check whether the choice of mapping program affected the reliability of chimeras, we performed more detailed analyses of the chimeras recovered with each program. The distributions of folding energies and counts of seed-mediated interactions were similar for all programs tested (Fig. 5a, b). All the programs identified similar sets of chimeras, and the majority of chimeras were identified by each of the mapping programs (Fig. 5c, d). When compared to the other read aligners, blat yielded slightly more chimeras with no gap or overlap between the two fragments, suggesting that it may be the method of choice when precise identification of ligation sites is required (not shown).

We also tested how the choice of mapping program influenced the degree to which the targets recovered for individual miRNAs are upregulated upon miRNA inhibition. We used two miRNA inhibition datasets: one with simultaneous depletion of 25 miRNAs [8] and one with depletion of miR-92a [11]. In each of the miRNA inhibition datasets, targets recovered by bowtie2 appeared to be most strongly upregulated, albeit the difference with the other programs is not statistically significant. However, cutoff e-value has an effect in this test (see below).

### 4.5 Optimization of chimera calling parameters

**Fig. 6:**
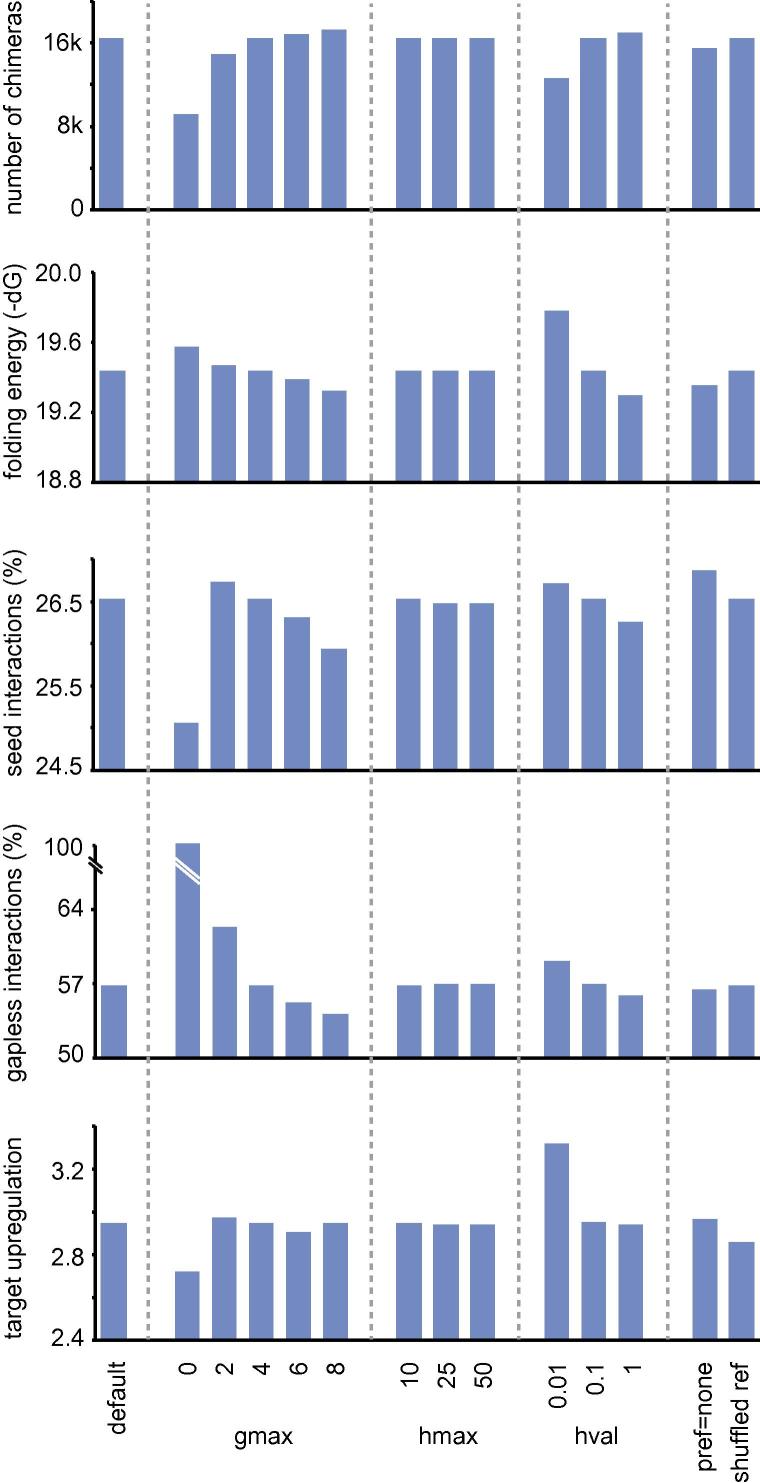
Benchmarking of chimera-calling options. Dataset E4 was analysed with default parameter values, except for the indicated options, which were individually changed. The resulting chimera counts and quality parameters are reported. The percent log2 transcript enrichment values used in the bottom panel are from [8].

We tested the impact of chimera calling options on the number and reliability of interactions recovered. Reliability was estimated from average folding energy, proportion of seed interactions, proportion of chimeras in which both fragments were directly adjacent in the read, and the extent of target upregulation upon mRNA depletion. Of all the parameters examined, the chimera e-value threshold (hval) had the largest effect (Fig. 6). Chimeras recovered with hval < 0.01 scored consistently higher than chimeras recovered with lower stringency. However, the number of chimeras recovered was lower by 20% with hval = 0.01, as compared to hval = 0.1. The optimum allowed gap or overlap between chimera fragments (gmax) appeared to be between 2 and 4. Lower or higher values tended to decrease the number of chimeras or decrease their reliability. Surprisingly, a cutoff of gmax = 0 also resulted in a lower fraction of seed interactions and lower upregulation of inferred targets. Disabling the mRNA–miRNA chimera preference decreased the number of called chimeras by 6%, but it did not consistently improve chimera quality. Disabling the preference of abundantly recovered transcripts (Section 3.3.3) had no effect on the number or quality of chimeras.

### 4.6 Influence of read and insert lengths

**Fig. 7:**
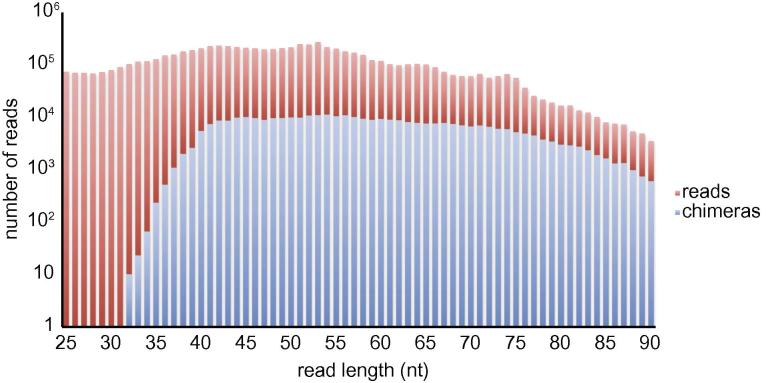
Numbers of chimeric and non-chimeric reads as a function of read length. Dataset E4 was analysed with default parameters.

Applying hyb to the data from Helwak et al. [11], we noticed that the same cDNA library (E4) yields a much larger fraction of chimeras when sequenced with 100 nucleotide-long reads than with 55-nt reads. This prompted us to examine the effect of RNA insert length on the recovery of chimeras. As expected, very few chimeras can be found in cDNAs of 35 nt or shorter, and the fraction of chimeras increases to more than ten percent when inserts of 80 nt or longer are considered (Fig. 7). The fraction of miRNA–mRNA chimeras among all reads also increases steadily with RNA insert length. We therefore recommend using 40 nt as minimum RNA insert length and 75 nt as the minimum read length in CLASH experiments.

### 4.7 Mapping chimeras with TopHat2 fusion

**Fig. 8:**
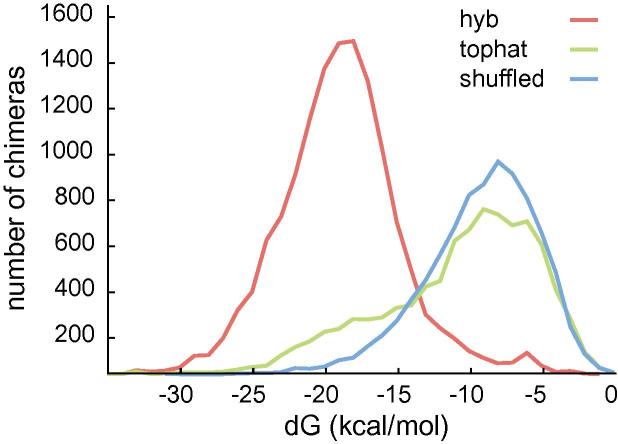
Comparison of hyb and tophat fusion. Distribution of folding energies of miRNA–mRNA chimeras recovered with hyb, tophat fusion, and in randomly re-associated miRNA–mRNA pairs from the tophat fusion analysis.

We compared hyb with the TopHat2 fusion-search algorithm, the most widely used tool for detecting fusion transcripts due to chromosomal rearrangements [28]. TopHat2’s fusion-search utilises Bowtie2, first storing initially unmapped reads which cannot be aligned to the genome end-to-end. These reads are cut into segments (default 25 bp), and individually aligned to the genome end-to-end. Reads with segments mapping to separate chromosomes, or further than 100 kb apart on the same chromosome are used to find the exact fusion point and create ‘spliced fusion contigs’. The initially unmapped reads are then re-aligned to these spliced fusion contigs with fusions being called if at least one alignment is spanning the read. We applied TopHat2 fusion-search to the E4 dataset from Helwak et al. [11] using segment lengths of 20 bp aligning to the human genome hg19. This identified 8231 microRNA–mRNA hybrids, in silico folding of which found the mean folding energy to be −10.3 kcal/mol, as compared with −8.7 kcal/mol with the miRNA–mRNA pairs were randomly re-associated (shuffled). By contrast, the mean folding energy of chimeras recovered with hyb was much lower: −18.2 kcal/mol from 13,493 hybrids (Fig. 8). This suggests that Tophat2 fusion identifies many chimeras that do not originate from RNA–RNA interactions. We hypothesize that many microRNA-containing reads are not aligned when Tophat2 cuts reads to fixed length segments followed by end-to-end mapping.

## 5 Application of hyb to CRAC, CLIP and RNA-Seq data

Chimeric reads corresponding to *bona fide* RNA–RNA interactions have previously been found in sequencing data generated by CRAC [12]. Although CRAC was not explicitly designed to ligate RNA–RNA hybrids and generate chimeras, we believe that chimeric RNAs are formed fortuitously during the 3′ linker addition step. This raises the question whether CLIP-Seq datasets might also contain chimeras indicative of RNA–RNA interaction. We have examined four AGO CLIP-Seq datasets [7–9,13], and found very few examples of microRNA–mRNA chimeras. There are several possible explanations why almost no chimeric reads are found in the CLIP-Seq data we analysed. First, reads used in each of the CLIP datasets are only 32–39 nt in length, which might preclude the identification of chimeras. Second, some CLIP protocols include polyacrylamide gel purification of AGO–RNA complexes before linker ligation. Non-crosslinked RNAs will most likely be lost during electrophoresis, transfer to nitrocellulose membrane, or proteinase K treatment of the membrane. Any of these events would result in the loss of RNA–RNA duplexes before chimeras can be formed. By contrast, in CRAC and CLASH protocols ligation precedes electrophoresis, making it possible to recover RNA–RNA duplexes as chimeric sequences.

In principle, hyb can also be applied to RNA-Seq data, and our initial analyses recovered significant numbers of chimeric sequences in RNA-Seq datasets. To assess the biological significance of such chimeras, we applied hyb to RNA-Seq data from HEK293 cells, used previously to discover circular RNAs [13]. Memczak et al. used an algorithm which identified head-to-tail (acceptor site followed by donor site) splice junctions by taking 20 bp from the termini of unaligned reads to map independently and screen for unique mappings which could be extended to cover the read with the breakpoint flanked by canonical splice sites. We applied hyb with alignment by blastall to reads annotated by Memczak et al. as covering 239 head-to-tail splice junctions. Aligning to the genome we identified 190 of these circular RNAs at the same location as Memczak et al. However, in aligning to our transcriptome database we identified reads corresponding to 32 circular RNAs which aligned to a transcript variant along at least 65-nucleotides of the 80-nucleotide read, such that the remaining read fragment could not be uniquely mapped. This suggests that some of the 239 candidate circular RNAs were variants of alternative splicing. Of the 26 circRNAs not identified as hybrid reads or seen with alignments across >65 nt, 5 were excluded due to gaps of >4 nucleotides between the alignments and the remaining 21 had better scoring individual alignments than each of those which would form a hybrid, and are therefore not called by hyb. We conclude that hyb can be used to reliably identify chimeric reads in CLASH, as well as in other next-generation sequencing datasets.

## Figures and Tables

**Table 1:** External dependencies of the hyb pipeline.

Package name	Reference	URL	Programs used
FASTX Toolkit	[30]	http://hannonlab.cshl.edu/fastx_toolkit/	Fastq_quality_trimmer
			Fastq_quality_filter
			Fastx_clipper
Flexbar	[17]	http://sourceforge.net/projects/flexbar/	Flexbar
FastQC	[16]	http://www.bioinformatics.babraham.ac.uk/projects/fastqc/	Fastqc
BLAST	[19]	http://www.ncbi.nlm.nih.gov/guide/data-software/	Blastall (legacy BLAST)
			Blastn (BLAST+)
BLAT	[31]	http://users.soe.ucsc.edu/~kent/src/blatSrc35.zip	Blat
PBLAT	[32]	http://code.google.com/p/pblat/	Pblat
Bowtie2	[18]	http://bowtie-bio.sourceforge.net/bowtie2/	Bowtie2
Unafold	[20]	http://mfold.rna.albany.edu/	Hybrid-min
Vienna	[21]	http://www.tbi.univie.ac.at/~ronny/RNA/RNAup.html	RNAup

**Table 2:** Default settings used for read alignment.

Program	Command line arguments
Blastall	-p blastn -W 11 -e 0.1 -m 8
Blastn	-Evalue 0.1 -num_threads 16 -word_size 11 -outfmt 6
Blat	-Stepsize = 5 -tileSize = 11 -minScore = 15 -out = blast8
Pblat	-Stepsize = 5 -tileSize = 11 -minScore = 15 -out = blast8
Bowtie2	-D 20 -R 3 -N 0 -L 16 -k 20 --local -i S,1,0.50 --score-min L,18,0 --ma 1 --np 0 --mp 2,2 --rdg 5,1 --rfg 5,1

**Table 3:** Example .hyb dataset.

44480_39	TGAGGTAG...	−12.1	let-7b	1	22	1	22	2e-04	ACTB	22	44	1614	1636	4e-05
72356_23	TGAGGTAG...	−21.2	let-7a	1	22	1	22	2e-04	CCNB2	23	49	399	425	2e-07
77750_22	ACTCTGTC...	−21.2	EIF2C1	1	27	1882	1908	2e-07	let-7a	28	49	1	22	2e-04
160269_10	TCCAACCT...	−18.7	EIF2C1	1	20	1888	1907	0.003	let-7a	21	42	1	22	2e-04
175997_9	TGAGGTAG...	−23	let-7b	1	22	1	22	3e-04	BAZ1B	23	60	916	953	8e-14
176006_9	TGAGGTAG...	−16.8	let-7a	1	22	1	22	2e-04	WIPI2	21	47	2704	2730	2e-07
180999_9	GTGAGGTA...	−12.1	let-7a	2	21	1	20	0.003	ZNF629	22	47	3264	3289	8e-07
193821_9	AATTCCCC...	−14.2	ARPP19	1	26	4702	4727	8e-07	let-7b	27	48	1	22	2e-04
199610_8	TCTGTCCA...	−21.2	EIF2C1	1	24	1884	1907	1e-05	let-7a	25	46	1	22	2e-04
211629_8	CTCTGTCC...	−21.2	EIF2C1	1	25	1883	1907	3e-06	let-7a	26	47	1	22	2e-04
220663_8	AGCCACCA...	−16	BAG4	1	41	546	586	1e-15	let-7b	42	63	1	22	3e-04
220664_8	AGCCACCA...	−17.8	NME4	1	29	724	752	1e-08	let-7b	30	51	1	22	2e-04
221833_8	ACTCTGTC...	−21.2	EIF2C1	1	26	1882	1907	8e-07	let-7a	27	48	1	22	2e-04
224142_8	AATCCACA...	−17.6	IPO7	1	26	2519	2544	8e-07	let-7b	27	48	1	22	2e-04
226595_8	AAACCAGA...	−18.3	SNUPN	1	21	663	683	7e-04	let-7c	22	43	1	22	2e-04
228636_7	TTCCAATA...	−16	LTA4H	1	28	655	682	6e-08	let-7a	29	50	1	22	2e-04
231753_7	TGAGGTAG...	−22.2	let-7b	1	22	1	22	2e-04	TSPAN3	23	49	1175	1201	2e-07
231779_7	TGAGGTAG...	−18.9	let-7e	1	22	1	22	2e-04	NCBP1	22	48	1657	1683	2e-07
231783_7	TGAGGTAG...	−21.6	let-7e	1	22	1	22	2e-04	RPL27A	20	40	1334	1354	7e-04
236865_7	TCCAACCT...	−18.7	EIF2C1	1	21	1888	1908	7e-04	let-7a	22	43	1	22	2e-04

Column 1, unique sequence identifier. Column 2, read sequence (truncated here for clarity). Column 3, predicted binding energy in kcal/mol. Columns 4–9, mapping information for first fragment of read: name of matched transcript, coordinates in read, coordinates in transcript, mapping score. Columns 10–15, mapping information for second fragment of read. Column 16 (optional, not shown here), list of annotations in the format: “feature1=value1; feature2=value2;..."
